# Combined analysis of gestational diabetes and maternal weight status from pre-pregnancy through post-delivery in future development of type 2 diabetes

**DOI:** 10.1038/s41598-021-82789-x

**Published:** 2021-03-03

**Authors:** Ling-Wei Chen, Shu E Soh, Mya-Thway Tint, See Ling Loy, Fabian Yap, Kok Hian Tan, Yung Seng Lee, Lynette Pei-Chi Shek, Keith M. Godfrey, Peter D. Gluckman, Johan G. Eriksson, Yap-Seng Chong, Shiao-Yng Chan

**Affiliations:** 1 Singapore Institute for Clinical Sciences, Agency for Science, Technology and Research, 30 Medical Drive, Singapore, 117609 Singapore; 2 Department of Paediatrics, Yong Loo Lin School of Medicine, National University of Singapore, 1E Kent Ridge Road, Singapore, 119228 Singapore; 3 Department of Obstetrics and Gynaecology, Yong Loo Lin School of Medicine, National University of Singapore, NUHS Tower Block, Level 12, 1E Kent Ridge Road, Singapore, 119228 Singapore; 4 Department of Reproductive Medicine, KK Women’s and Children’s Hospital, 100 Bukit Timah Road, Singapore, 229899 Singapore; 5 Duke-National University of Singapore Graduate Medical School, 8 College Road, Singapore, 169857 Singapore; 6 Department of Pediatric Endocrinology, KK Women’s and Children’s Hospital, 100 Bukit Timah Road, Singapore, 229899 Singapore; 7 Department of Maternal Fetal Medicine, KK Women’s and Children’s Hospital, 100 Bukit Timah Road, Singapore, 229899 Singapore; 8 Khoo Teck Puat-National University Children’s Medical Institute, National University Health System, 1E Kent Ridge Road, Singapore, 119228 Singapore; 9 MRC Lifecourse Epidemiology Unit & NIHR Southampton Biomedical Research Centre, University of Southampton & University Hospital Southampton NHS Foundation Trust, Tremona Road, Southampton, SO16 6YD UK; 10 Liggins Institute, University of Auckland, 85 Park Rd, Grafton, Auckland, 1023 New Zealand; 11 Department of General Practice and Primary Health Care, University of Helsinki, Haartmaninkatu 8, 00290 Helsinki, Finland; 12 Folkhälsan Research Center, Topeliusgatan 20, 00250 Helsinki, Finland

**Keywords:** Diseases, Endocrinology, Health care, Medical research, Risk factors

## Abstract

We examined the associations of gestational diabetes mellitus (GDM) and women’s weight status from pre-pregnancy through post-delivery with the risk of developing dysglycaemia [impaired fasting glucose, impaired glucose tolerance, and type 2 diabetes (T2D)] 4–6 years post-delivery. Using Poisson regression with confounder adjustments, we assessed associations of standard categorisations of prospectively ascertained pre-pregnancy overweight and obesity (OWOB), gestational weight gain (GWG) and substantial post-delivery weight retention (PDWR) with post-delivery dysglycaemia (*n* = 692). Women with GDM had a higher risk of later T2D [relative risk (95% CI) 12.07 (4.55, 32.02)] and dysglycaemia [3.02 (2.19, 4.16)] compared with non-GDM women. Independent of GDM, women with pre-pregnancy OWOB also had a higher risk of post-delivery dysglycaemia. Women with GDM who were OWOB pre-pregnancy and had subsequent PDWR (≥ 5 kg) had 2.38 times (1.29, 4.41) the risk of post-delivery dysglycaemia compared with pre-pregnancy lean GDM women without PDWR. No consistent associations were observed between GWG and later dysglycaemia risk. In conclusion, women with GDM have a higher risk of T2D 4–6 years after the index pregnancy. Pre-pregnancy OWOB and PDWR exacerbate the risk of post-delivery dysglycaemia. Weight management during preconception and post-delivery represent early windows of opportunity for improving long-term health, especially in those with GDM.

## Introduction

Diabetes is a major global health threat that accounted for 4.2 million deaths worldwide in 2019^1^. The global prevalence of diabetes has risen from 4.7% in 1980^2^ to 9.3% in 2019, and is projected to reach 10.9% by 2045^1^. Globally it is estimated that one in seven pregnancies is affected by gestational diabetes mellitus (GDM)^3^. GDM may be an antecedent to later type 2 diabetes (T2D); women with a history of GDM were about 7–9 times as likely to develop T2D compared to those without GDM in meta-analyses^4–6^. These meta-analyses included many studies utilising older criteria for GDM diagnosis, which identified patients at the more severe end of the GDM spectrum. However, the Hyperglycemia and Adverse Pregnancy Outcome (HAPO) study, from which the present International Association of Diabetes and Pregnancy Study Groups (IADPSG) GDM diagnostic criteria were derived, showed that less severe maternal hyperglycaemia was also associated with elevated risks of adverse pregnancy outcomes^7^ and development of T2D or prediabetes at a median follow-up of 11.4 years^8^. This highlights the detrimental consequences of even milder forms of GDM based on contemporary criteria^9^. Patient education, lifestyle interventions and regular screening for pre-diabetes after a pregnancy complicated by GDM offer a window of opportunity to prevent or delay onset of T2D.

Weight appears to be a key prognostic factor and mediator in the development of T2D following a GDM pregnancy. Previous meta-analyses involving mostly studies in European and North American populations suggested that maternal overweight/obesity and increased gestational weight gain are associated with a higher risk of GDM^10,11^. It was unclear if an elevated weight acts as a confounder or adds additional risk to the development of future T2D following a GDM-complicated pregnancy. One study showed that among Chinese women with a history of GDM, pre-pregnancy obesity and substantial post-delivery weight gain elevated the risk for developing T2D and prediabetes at 1–5 years after delivery^12^; however, this study did not investigate gestational weight gain nor quantify the additive risks of all these factors (GDM and weight status) combined. An intensive lifestyle intervention, with weight loss being a critical element, in women with a history of GDM reduced the occurrence of T2D within 3 years of delivery by 50%^13^. Many studies have explored separately the impact of pre-pregnancy body mass index (BMI), trimester-specific gestational weight gain^14,15^ and post-delivery weight retention on GDM and T2D risk^16–18^. However, weight status and weight-change of women from pre-conception, through pregnancy and to years after delivery should be considered in combination to identify critical window periods to target for most cost-effective intervention.

We hypothesised that an elevated pre-pregnancy BMI alongside excessive gestational weight gain and postpartum weight retention will further exacerbate the risk of developing future T2D and prediabetes following a pregnancy complicated by GDM. Using data from a prospective Asian mother–offspring cohort in Singapore, Growing Up in Singapore Towards healthy Outcomes (GUSTO), the aims of the current study were two-fold. First, we aimed to describe, for the first time in a general multi-ethnic Asian population who were universally screened for GDM, the incidence of new onset pre-diabetes and T2D within 4–6 years of an index pregnancy complicated by GDM, compared to those who had normal glucose tolerance in pregnancy. Second, we investigated the prospective relationships of maternal weight and BMI status from pre-pregnancy through post-delivery with new onset prediabetes and T2D, and whether they exacerbate the influence of GDM.

## Methods

### Study participants

A total of 1450 participants were recruited into the on-going GUSTO mother–offspring cohort study (ClinicalTrials.gov identifier: NCT01174875), which studies the impact of gene–environment interaction on long-term maternal and child health^19^. Pregnant women aged 18 years and above were recruited at < 14 weeks gestation from two main public maternity hospitals in Singapore. The Chinese, Malay or Indian participants were Singapore citizens or permanent residents. Mothers receiving chemotherapy, psychotropic drugs or who had type 1 diabetes mellitus were excluded. Mothers with possible pre-existing T2D and chronic hypertension were not excluded at the outset, but we conducted sensitivity analyses excluding these participants in the current study. The design of the study has been detailed elsewhere^19^. This study was approved by the National Health Care Group Domain Specific Review Board (reference D/09/021) and the SingHealth Centralized Institutional Review Board (reference 2009/280/D). All research was performed in accordance with the relevant guidelines and informed consent was obtained from all participants upon recruitment.

### Maternal data

Ethnicity, educational attainment, family history of diabetes were self-reported at study enrolment. Maternal age at delivery was calculated from the date of birth retrieved from national registration and the date of delivery. Parity, personal history of chronic hypertension and pregnancy-induced hypertension (including pre-eclampsia and non-proteinuric pregnancy-induced hypertension) were abstracted from medical records. Cigarette smoking, breastfeeding duration and medical history were obtained through interviewer-administered questionnaires.

### Ascertainment of GDM and dysglycaemia after delivery

Both GDM during pregnancy and dysglycaemia at 4–6 years post-delivery were diagnosed using a 2-h (2 h) 75 g oral glucose tolerance test (OGTT) after an overnight fast. GDM was defined by the WHO 1999 criteria which was in use at the time of the study (fasting glucose ≥ 7.0 mmol/L and/or 2 h glucose ≥ 7.8 mmol/L). Any dysglycaemia at 4–6 years post-delivery were defined as having pre-diabetes [impaired fasting glucose (IFG, fasting glucose 6.0–6.9 mmol/L), impaired glucose tolerance (IGT, 2 h glucose 7.8–11.0 mmol/L)] or type 2 diabetes mellitus (T2D; fasting glucose ≥ 7.0 mmol/L and/or 2 h glucose ≥ 11.1 mmol/L) (see Supplemental Table 1)^20,21^. T2D was also investigated as a separate outcome.

### Maternal anthropometry

Pre-pregnancy weight was self-reported at recruitment. Routinely measured weights during pregnancy at up to nine time-points spanning the first to the last antenatal visit were abstracted from the medical records. Additionally, maternal weight and height at 26–28 weeks’ gestation were measured in duplicates using SECA 803 Weighing Scale and SECA 213 Stadiometer (SECA Corp, Hamburg, Germany) by trained research staff. Similarly, after delivery, weight and height were measured by trained research staff at 18 months and 4 years post-delivery.

#### Pre-pregnancy BMI

Pre-pregnancy BMI was calculated by dividing participants’ self-reported pre-pregnancy weight in kilogram (kg) by the participants’ measured height in meter-squared (m^2^). Participants were then categorized as being underweight (< 18.5 kg/m^2^), normal weight (18.5–22.9 kg/m^2^), overweight (23.0–27.4 kg/m^2^), or obese (≥ 27.5 kg/m^2^) using established Asian cut-offs^22,23^.

#### Gestational weight gain (GWG)

Participants were classified into groups of inadequate, adequate and excessive weight gain based on the Institute of Medicine (IOM) recommended absolute weight gain (for total gestational weight gain) and rate of weight gain (kg/week) (for weight gain during second and third trimesters) according to pre-pregnancy BMI category (see Supplemental Table 2)^24,25^. Total gestational weight gain was computed by subtracting first antenatal visit weight from last antenatal visit weight. To compute rate of weight gain during second and third trimesters, linear mixed-effects model with the Best Linear Unbiased Predictor was used to estimate linear trajectory of GWG per week^26^. Because participants might have changed their lifestyle behaviors after GDM diagnosis (at approximately 26–28 weeks’ gestation), weight gain rates for periods before and after GDM diagnosis (or OGTT conduct in the case of non-GDM cases) were generated separately. For both weight gain rates, we only included participants with at least two weight measurements within the defined periods [(1) at or after 12 weeks’ gestational age but before OGTT/GDM diagnosis and (2) at or after OGTT/GDM diagnosis until delivery]. Inadequate GWG was defined as an absolute weight gain or weight gain rate less than the IOM recommended lower limit, whereas excessive weight gain was defined as absolute weight gain or weight gain rate greater than the recommended upper limit. Other participants with weight gain or weight gain rate within the recommended range were classified as having adequate GWG, the reference group in our analyses.

#### Post-delivery weight retention (PDWR) and BMI change

According to standard cut-off, PDWR was considered substantial when women gained, with reference to their pre-pregnancy weights, equal to or more than 5 kg in weight at 1–2 years after delivery^27,28^. Change in BMI categories was also considered, by categorising women into four groups: (1) lean (< 23 kg/m^2^) pre-pregnancy and remained lean after delivery (Lean–> Lean); (2) lean pre-pregnancy and becoming overweight or obese (OWOB; ≥ 23 kg/m^2^) after delivery (Lean–> OWOB); (3) OWOB pre-pregnancy and becoming lean after delivery (OWOB–> Lean); and (4) OWOB both pre-pregnancy and after delivery (OWOB–> OWOB).

### Statistical analyses

Descriptive statistics are reported as *n* (%) for categorical variables and means (SD) for continuous variables. Chi-square tests and independent *t*-tests were used to compare characteristics. All statistical tests were two sided and a *P* value < 0.05 was considered to be statistically significant.

The primary outcomes were dysglycaemia and T2D post-delivery while the exposures were GDM and weight status/gain/change from pre-pregnancy through post-delivery (i.e., pre-pregnancy BMI, GWG, PDWR). Relative risk (RR) and 95% confidence intervals (CI) of GDM and peri-pregnancy weight status with any dysglycaemia and T2D post-delivery were calculated using Poisson regression with robust standard errors. The regressions were conducted unadjusted and adjusted for important covariates based on existing literature: ethnicity, age at delivery, education (as a measure of socioeconomic status), parity, family history of diabetes, insulin treatment during pregnancy and pregnancy-induced hypertension. Apart from investigating the risk factors individually, we also modelled the risk for any dysglycaemia by looking at combinations of these risk factors (there were insufficient numbers for T2D modelling). No data imputation was undertaken for missing data and only cases with relevant datasets were included.

In the sensitivity analyses, we excluded (1) participants who had antenatal OGTT conducted < 24 weeks or > 32 weeks (*n* = 30; outside the window period conventionally used to define glycaemia thresholds in pregnancy) and with possible pre-existing T2D suggested by fasting glucose ≥ 7.0 mmol/L and/or 2 h glucose ≥ 11.1 mmol/L (*n* = 4) and (2) participants with chronic hypertension (*n* = 14; a common co-morbid condition of T2D) to confirm the consistency and robustness of association between GDM and post-delivery dysglycaemia. To assess if adoption of the newer criteria could potentially alter the relationships between combination of risk factors and future dysglycaemia, we also retrospectively applied the IADPSG criteria in a partial manner (GDM diagnosed by ≥ 5.1 mmol/L for fasting glucose and/or ≥ 8.5 mmol/L for 2 h glucose, without the 1-h [1 h] measure which was not performed at that time). All analyses were performed using Stata software (version 15.1, Statacorp, College Station, Texas).

## Results

Among recruited women, 1239 had singleton pregnancies and still remained in the study at 26–28 weeks’ gestation. Of these subjects, 1165 (94.0%) had antenatal OGTT results and 692 (59.4% of total *n* with antenatal OGTT) had both antenatal and post-delivery OGTT (see Supplemental Fig. 1 for participant flow chart). Characteristics of participants who had both antenatal and postnatal OGTT conducted (*n* = 692) with relevant covariate data and included in this study, were slightly older, more likely to be parous and less likely to have had pregnancy-induced hypertension compared with those who only had antenatal OGTT (*n* = 473) and therefore not included in this study (Supplemental Table 3). Ethnicity, family history of diabetes, smoking status, breastfeeding duration and peri-pregnancy BMI were comparable between the groups (Supplemental Table 3).

Among included subjects, 142 (20.5%) had GDM, of which 99.3% were diagnosed based on an elevated antenatal 2 h glucose measure alone. Table 1 describes the demographic and clinical characteristics of included participants according to GDM and post-delivery dysglycaemia (IFG/IGT/T2D) status. In the GDM group, the majority were Chinese (63.4%), followed by Indian (22.5%) and Malay (14.1%). The women with GDM were older, more likely to have pregnancy-induced hypertension and had higher BMI from preconception through to 26–28 weeks’ gestation (Table 1). Compared with participants with normal glucose tolerance post-delivery, participants with dysglycaemia 4–6 years post-delivery (18.6%) were older, had higher BMI from preconception to post-delivery, and more likely to have a family history of diabetes, and pregnancy-induced hypertension and insulin treatment for GDM in the index pregnancy (Table 1).

**Table 1:** Characteristics of participants according to GDM and post-delivery dysglycaemia status.

	Non-GDM	GDM	*P* value	Normal glucose tolerance	Dysglycaemia	*P* value
	*n* = 550	*n* = 142		*n* = 563	*n* = 129	
**Ethnicity**			0.003			0.25
Chinese	307 (55.8%)	90 (63.4%)		331 (58.8%)	66 (51.2%)	
Malay	152 (27.6%)	20 (14.1%)		137 (24.3%)	35 (27.1%)	
Indian	91 (16.5%)	32 (22.5%)		95 (16.9%)	28 (21.7%)	
**Highest educational attainment**			0.15			0.25
No education/primary/secondary	163 (30.0%)	31 (21.8%)		162 (29.0%)	32 (25.0%)	
Post-secondary/pre-university	188 (34.6%)	53 (37.3%)		188 (33.7%)	53 (41.4%)	
University	193 (35.5%)	58 (40.8%)		208 (37.3%)	43 (33.6%)	
**Age at delivery (years)**	31.3 (4.9)	33.5 (4.6)	< 0.001	31.5 (4.9)	32.8 (5.0)	0.008
**Parity at index pregnancy**			0.92			0.86
Nulliparous	239 (43.5%)	61 (43.0%)		245 (43.5%)	55 (42.6%)	
Parous	311 (56.5%)	81 (57.0%)		318 (56.5%)	74 (57.4%)	
**Family history of diabetes**			0.60			0.012
No	376 (69.9%)	96 (67.6%)		395 (71.6%)	77 (60.2%)	
Yes	162 (30.1%)	46 (32.4%)		157 (28.4%)	51 (39.8%)	
**Insulin treatment for GDM**			–			< 0.001
No	–	132 (93.0%)		561 (99.6%)	121 (93.8%)	
Yes	–	10 (7%)		2 (0.4%)	8 (6.2%)	
**Hypertension before index pregnancy**			0.23			0.64
No	545 (99.1%)	139 (97.9%)		557 (98.9%)	127 (98.4%)	
Yes	5 (0.9%)	3 (2.1%)		6 (1.1%)	2 (1.6%)	
**Pregnancy-induced hypertension in index pregnancy**			0.029			0.003
No	528 (96.0%)	130 (91.5%)		542 (96.3%)	116 (89.9%)	
Yes	22 (4.0%)	12 (8.5%)		21 (3.7%)	13 (10.1%)	
**Hypertension after index pregnancy**			0.15			0.22
No	533 (96.9%)	134 (94.4%)		545 (96.8%)	122 (94.6%)	
Yes	17 (3.1%)	8 (5.6%)		18 (3.2%)	7 (5.4%)	
**Maternal smoking during pregnancy**			0.30			0.055
No	525 (96.0%)	136 (97.8%)		534 (95.7%)	127 (99.2%)	
Yes	22 (4.0%)	3 (2.2%)		24 (4.3%)	1 (0.8%)	
**Any breastfeeding beyond 6 months**			0.94			0.17
No	314 (59.4%)	80 (59.7%)		316 (58.2%)	78 (65.0%)	
Yes	215 (40.6%)	54 (40.3%)		227 (41.8%)	42 (35.0%)	
**Pre-pregnancy maternal BMI**	22.5 (4.3)	23.9 (4.5)	< 0.001	22.2 (4.0)	25.3 (5.0)	< 0.001
**Pregnancy maternal BMI (booking)**	23.4 (4.7)	24.7 (4.5)	0.004	23.1 (4.4)	26.4 (5.2)	< 0.001
**Pregnancy maternal BMI (26–28 wks)**	26.0 (4.4)	27.2 (4.1)	0.003	25.7 (4.1)	28.3 (4.8)	< 0.001
**Pregnancy maternal BMI (last antenatal visit)**	28.1 (4.4)	28.5 (4.3)	0.30	27.8 (4.2)	30.1 (4.9)	< 0.001
**Postpartum maternal BMI (18 months post-delivery)**	24.0 (4.8)	24.6 (4.4)	0.20	23.5 (4.4)	26.8 (4.9)	< 0.001
**Postpartum maternal BMI (4 years post-delivery)**	24.6 (5.3)	25.1 (4.7)	0.27	24.0 (4.8)	27.6 (5.8)	< 0.001

*GDM* gestational diabetes mellitus, *BMI* body mass index, *wks* weeks’ gestation. Data are presented as *n *(%) for categorical variables or mean (SD) for continuous variables.

### GDM and post-delivery dysglycaemia

In both unadjusted and adjusted analyses, GDM was associated with a significantly higher risk of having any dysglycaemia (IFG/IGT/T2D) and T2D post-delivery. Among mothers with a GDM-complicated pregnancy, 43.4% developed dysglycaemia at 4–6 years post-delivery, as compared to 12.3% for mothers without GDM (Table 2). After adjusting for covariates and when compared to women without a GDM diagnosis during the index pregnancy, women with a GDM-complicated pregnancy had three times the risk of dysglycaemia [adjusted relative risk (aRR): 3.02 (95% CI 2.19, 4.16)] and 12 times the risk of T2D [aRR: 12.07 (4.55, 32.02)] (Table 2). When participants who had antenatal OGTT conducted at < 24 weeks’ or > 32 weeks’ gestation or with possible pre-existing T2D were excluded, similar associations remained [aRR: 3.08 (2.19, 4.33) for dysglycaemia and 13.43 (4.97, 36.26) for T2D; both *P* < 0.001]. Similarly, results remained highly statistically significant with exclusion of participants with chronic hypertension [aRR: 2.94 (2.12, 4.06) for dysglycaemia and 10.29 (3.73, 28.41) for T2D; both *P* < 0.001].

**Table 2:** Prospective associations of gestational diabetes and weight status from pre-pregnancy through post-delivery periods, with dysglycaemia and type 2 diabetes risks at 4–6 years post-delivery.

	Case/total (%)	Dysglycaemia (IFG/IGT/T2D)		Case/total (%)	Type 2 diabetes	
		Unadjusted	Adjusted^a^		Unadjusted	Adjusted^a^
		RR (95% CI)^b^	RR (95% CI)^b^		RR (95% CI)^b^	RR (95% CI)^b^
**Gestational diabetes mellitus (GDM)**						
No	68/550 (12.3%)	Ref	Ref	5/550 (0.9%)	Ref	Ref
Yes	62/142 (43.4%)	3.47 (2.59, 4.66)***	3.02 (2.19, 4.16)***	18/142 (12.6%)	13.84 (5.26, 36.94)***	12.07 (4.55, 32.02)***
**Weight status**						
(A) Pre-pregnancy BMI						
Underweight	7/73 (9.6%)	0.78 (0.37, 1.68)	0.90 (0.42, 1.94)	4/392 (1.0%)	Ref.^c^	Ref.^c^
Normal	39/319 (12.2%)	Ref	Ref			
Overweight	42/159 (26.4%)	2.16 (1.46, 3.20)***	2.01 (1.34, 3.02)**	8/159 (5.0%)	4.93 (1.50, 16.16)**	3.77 (1.13, 12.62)*
Obese	38/93 (40.9%)	3.34 (2.28, 4.90)***	2.85 (1.82, 4.47)***	12/93 (12.9%)	12.65 (4.17, 38.36)***	7.20 (1.91, 27.14)**
(B) Gestational weight gain (GWG)						
Total GWG						
Inadequate	52/243 (21.3%)	1.11 (0.78, 1.58)	1.21 (0.86, 1.72)	12/243 (4.9%)	2.01 (0.77, 5.27)	3.03 (1.03, 8.92)*
Adequate	47/244 (19.3%)	Ref	Ref	6/244 (2.5%)	Ref	Ref
Excessive	22/129 (17.1%)	0.89 (0.56, 1.40)	0.85 (0.54, 1.34)	6/129 (4.7%)	1.89 (0.62, 5.75)	2.54 (0.82, 7.88)
GWG rate before OGTT						
Inadequate	18/82 (22.0%)	1.40 (0.85, 2.31)	1.50 (0.91, 2.47)	4/82 (4.9%)	2.43 (0.67, 8.84)	2.24 (0.58, 8.57)
Adequate	39/249 (15.6%)	Ref	Ref	5/249 (2.0%)	Ref	Ref
Excessive	51/231 (22.1%)	1.41 (0.97, 2.05)	1.22 (0.83, 1.80)	12/231 (5.2%)	2.59 (0.92, 7.24)	2.00 (0.68, 5.92)
GWG rate after OGTT						
Inadequate	33/170 (19.4%)	1.10 (0.71, 1.69)	1.15 (0.76, 1.74)	3/170 (1.8%)	0.42 (0.11, 1.57)	0.47 (0.14, 1.61)
Adequate	34/192 (17.7%)	Ref	Ref	8/192 (4.2%)	Ref	Ref
Excessive	46/222 (20.7%)	1.17 (0.78, 1.74)	1.04 (0.69, 1.56)	9/222 (4.1%)	0.97 (0.38, 2.47)	0.95 (0.35, 2.61)
(C) Post-delivery weight retention (PDWR)/ BMI change						
PDWR at 18 months						
Non substantial	65/361 (18.0%)	Ref	Ref	14/361 (3.9%)	Ref	Ref
Substantial	35/148 (23.7%)	1.31 (0.91, 1.89)	1.26 (0.87, 1.84)	6/148 (4.1%)	1.06 (0.41, 2.67)	1.03 (0.41, 2.63)
BMI change at 18 months						
Lean–> lean	20/227 (8.8%)	Ref	Ref	3/227 (1.3%)	Ref	Ref
Lean–> OWOB	14/74 (18.9%)	2.15 (1.14, 4.03)*	2.03 (1.09, 3.77)*	0/74 (0.0%)	NA	NA
OWOB–> lean	2/9 (22.2%)	2.52 (0.69, 9.19)	2.11 (0.63, 7.03)	1/9 (11.1%)	8.41 (0.97, 73.24)	1.94 (0.31, 11.97)
OWOB–> OWOB	63/189 (33.3%)	3.78 (2.38, 6.02)***	3.13 (1.90, 5.14)***	15/189 (7.9%)	6.01 (1.76, 20.46)**	3.75 (1.01, 13.97)*
PDWR at 4 years						
Non-substantial	68/340 (17.1%)	Ref	Ref	15/340 (4.4%)	Ref	Ref
Substantial	56/234 (23.9%)	1.40 (1.01, 1.95)*	1.47 (1.05, 2.05)*	8/234 (3.4%)	0.77 (0.33, 1.80)	0.84 (0.32, 2.16)
BMI change at 4 years						
Lean–> lean	24/244 (9.8%)	Ref	Ref	3/244 (1.2%)	Ref	Ref
Lean–> OWOB	15/96 (15.6%)	1.59 (0.87, 2.90)	1.58 (0.88, 2.83)	1/96 (1.0%)	0.85 (0.09, 8.06)	0.70 (0.07, 6.69)
OWOB–> lean	3/9 (33.3%)	3.39 (1.25, 9.21)*	3.18 (1.23, 8.21)*	1/9 (11.1%)	9.04 (1.04, 78.76)*	2.69 (0.51, 14.16)
OWOB–> OWOB	71/212 (33.5%)	3.40 (2.23, 5.21)***	2.97 (1.89, 4.67)***	18/212 (8.5%)	6.91 (2.06, 23.14)**	4.17 (1.19, 14.67)*

*IFG* impaired fasting glucose, *IGT* impaired glucose tolerance, *T2D* type 2 diabetes, *RR* relative risk, *Ref.* reference, *BMI* body mass index, *GDM* gestational diabetes mellitus, *GWG* gestational weight gain, *PDWR* post-delivery weight retention, *OWOB* overweight or obese.

*P < 0.05; **P < 0.01; ***P < 0.001.

^a^Adjusted for ethnicity, age at delivery, education, parity, family history of diabetes, insulin treatment during pregnancy and pregnancy induced hypertension.

^b^Estimates are relative risk (95% CI) for dysglycaemia and type 2 diabetes post-delivery according to the studied exposure.

^c^Due to insufficient cases of T2D in underweight categories and because the relationship between pre-pregnancy BMI and dysglycaemia appeared linear, we combined underweight and normal weight categories into reference group for this analysis.

### Pre-pregnancy BMI and post-delivery dysglycaemia

Compared to normal weight women, women who were overweight and obese pre-pregnancy had a significantly higher risk of developing any dysglycaemia and T2D post-delivery. There was a gradation of effect with increasing BMI. In adjusted models, overweight and obese women had approximately two times and three times the risk (both *P* < 0.01), respectively, of developing dysglycaemia compared to normal weight mothers (Table 2). These associations were independent of GDM diagnosis, as both pre-pregnancy overweight and GDM remained statistically significant risk factors for dysglycaemia when they were mutually adjusted for (results not shown). The relative risks of developing T2D were even greater; almost four times for overweight and seven times for obese women (Table 2).

### Gestational weight gain (GWG) and post-delivery dysglycaemia

Overall, we did not observe any consistent association between total GWG, GWG rate before or after GDM diagnosis, with the risk of developing any dysglycaemia or T2D post-delivery (Table 2). An exception was noted for inadequate total GWG, which was associated with a higher risk of T2D [RR (95% CI) 3.03 (1.03, 8.92)] compared with adequate total GWG (Table 2).

### Post-delivery weight retention (PDWR), BMI change and post-delivery dysglycaemia

PDWR (≥ 5 kg with reference to pre-pregnancy weight) at 4 years post-delivery was associated with 1.5 times the risk of dysglycaemia; no consistent associations were observed for PDWR at 18 months. However, when weight change was categorised according to pre-pregnancy and post-delivery lean (< 23 kg/m^2^) and overweight/obese (OWOB; ≥ 23 kg/m^2^) status, women who were OWOB pre-pregnancy and remained OWOB at 18 months or 4 years post-delivery had consistently higher risk of developing any dysglycaemia (approximately three times) and T2D (approximately four times), as compared with women who were lean at both time-points (Table 2). Moreover, albeit based on small numbers, participants who transitioned from pre-pregnancy lean to post-delivery OWOB at 18 months also showed an increased risk of dysglycaemia. Also, despite transitioning from pre-pregnancy OWOB to post-delivery lean at 4 years there remained a higher risk of post-delivery dysglycaemia (Table 2).

### Combinations of risk factors and post-delivery dysglycaemia

**Figure 1:**
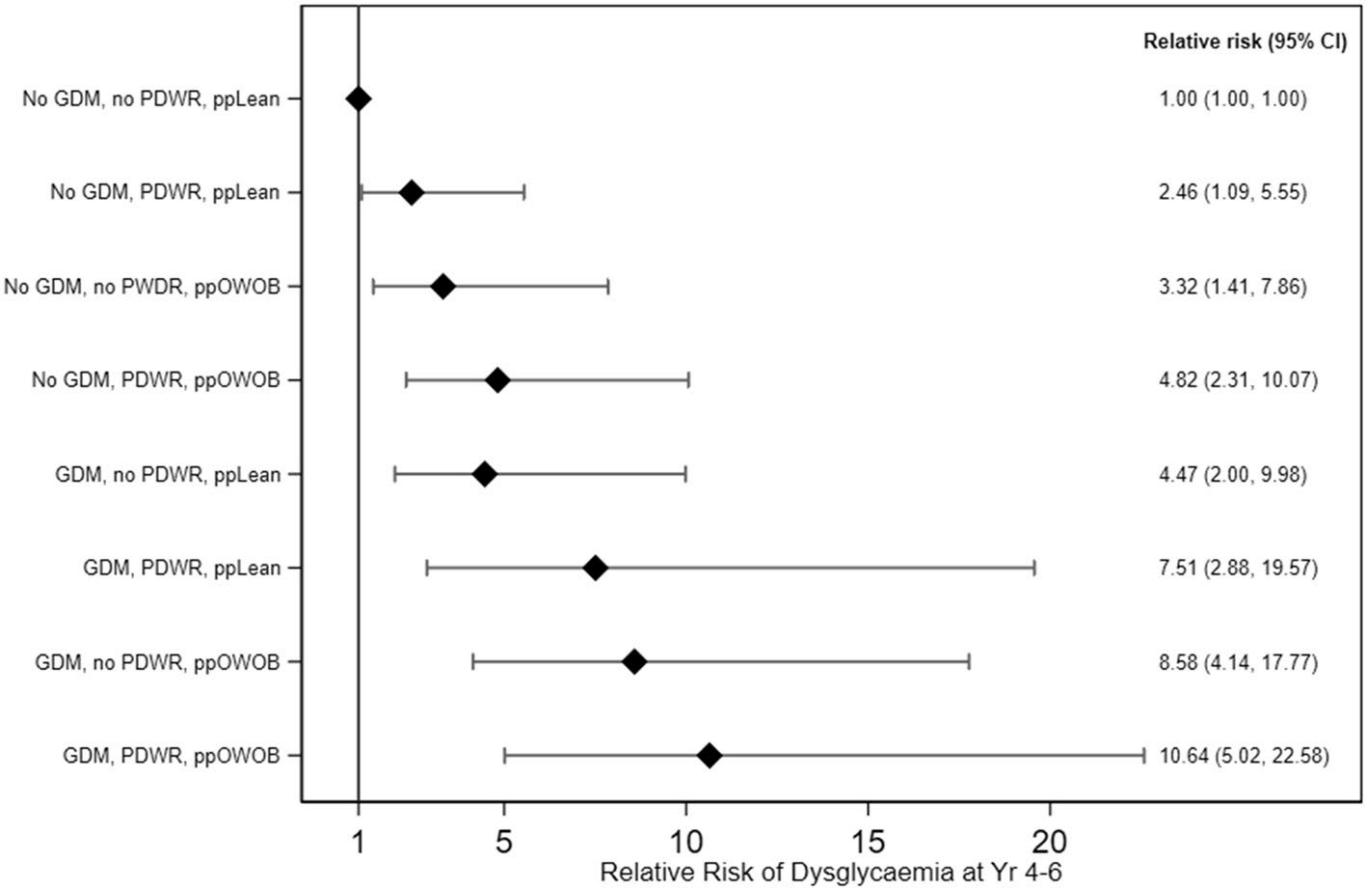
Relative risk of dysglycaemia at 4–6 years post-delivery according to combinations of peri-pregnancy risk factors. The diamonds and capped lines represent point estimates and 95% confidence intervals, respectively, of relative risk of having dysglycaemia at 4–6 years post-delivery according to combinations of peri-pregnancy risk factors. Reference group comprised participants without any of the risk factor. Estimates are adjusted for ethnicity, age at delivery, education, parity, family history of diabetes, insulin treatment during pregnancy and pregnancy induced hypertension. *GDM* gestational diabetes mellitus, *PDWR* post-delivery weight retention (≥ 5 kg) at 4 years, *ppLean* pre-pregnancy lean (BMI < 23 kg/m^2^), *ppOWOB* pre-pregnancy overweight or obese (BMI ≥ 23 kg/m^2^).

We further investigated the combined influence of GDM, substantial PDWR, and pre-pregnancy lean/OWOB status on dysglycaemia. Participants with the lowest risk (i.e. non-GDM, no substantial PDWR at 4 years, and pre-pregnancy lean) were used as the reference group. Compared to this reference group, substantial PDWR alone (in pre-pregnancy lean and non-GDM participants) was associated with 2.46 times (95% CI 1.09, 5.55) the risk of dysglycaemia at 4–6 years post-delivery; the risk was further doubled [4.82 (2.31, 10.07)] if participants had also been OWOB pre-pregnancy in addition to having substantial PDWR (Fig. 1). GDM alone (lean and without substantial PDWR) demonstrated 4.47 times (2.00, 9.98) the risk of dysglycaemia compared with the reference, a magnitude similar to that of the non-GDM group with both the two other risk factors (i.e. pre-pregnancy OWOB and substantial PDWR). Having these further two risk factors on top of GDM incrementally increased the relative risk for post-delivery dysglycaemia. In participants with all the three risk factors, the risk of developing dysglycaemia 4–6 years post-delivery was 10.64 times as high (5.02, 22.58) compared to participants with none of the risk factors (Fig. 1). When the reference group was changed to GDM participants without PDWR and who were lean pre-pregnancy, GDM participants with PDWR and who were OWOB pre-pregnancy had an adjusted relative risk of 2.38 (1.29, 4.41) of developing post-delivery dysglycaemia, indicating that having PDWR and pre-pregnancy OWOB exacerbated the adverse influence of GDM. In sensitivity analysis with GDM defined using partial IADPSG criteria without the 1 h glucose measure, the overall trends of a higher risk with an increasing number of risk factors, as compared to participants without any risk factors, were similar, but the effect estimates were attenuated (see Supplemental Fig. 2).

## Discussion

In this multi-ethnic Asian prospective cohort, women who had a GDM-complicated pregnancy had 12 times the risk of developing T2D within 4–6 years after the index pregnancy compared to non-GDM cases. Overall, 43.4% of women who had GDM developed dysglycaemia within 4–6 years post-delivery, representing a substantial proportion who required clinical management. Independent of GDM but to a lesser extent, pre-pregnancy OWOB and (separately) substantial PDWR also increased the risk of dysglycaemia post-delivery when compared to the lowest risk group. Although each of these risk factors (GDM, pre-pregnancy OWOB, and substantial PDWR) incrementally increased risk of dysglycaemia, having GDM alone contributed equivalent risk to the combination of having pre-pregnancy OWOB and substantial PDWR without GDM. The highest risk was observed when all three risk factors were present, with ten times the risk of post-delivery dysglycaemia compared to those with none of these risk factors. No consistent associations were observed between GWG and post-delivery dysglycaemia. To reduce the risk of long-term dysglycaemia our study highlights the need for a combination of public health messaging to maintain BMI in a healthy range prior to pregnancy combined with weight management interventions (such as improvement of diet and increased physical activity)^29^ after pregnancy, especially in those who had pregnancies complicated by GDM.

Our work contributes to the existing knowledge on the development of type 2 diabetes after a GDM-complicated pregnancy^4^, particularly amongst multi-ethnic Asian women. In accord with published observations^17,30,31^, women with a history of GDM in our cohort demonstrated a high incidence of impaired glucose regulation (43.4%, of which 12.6% were consistent with new onset T2D) within a relatively short time period of 4–6 years after delivery. This increased risk of T2D [unadjusted and adjusted RR (95% CI) 13.84 (5.26, 36.94) and 12.07 (4.55, 32.02), respectively] is higher than those reported in the meta-analysis by Bellamy et al.^4^ [pooled unadjusted RR (95% CI) 7.43 (4.79, 11.51)] and Vounzoulaki et al.^6^ [pooled adjusted RR 9.51 (7.14–12.67)], which included studies conducted over longer periods of time, up to 28 years after delivery, and included many studies performed in White Caucasian and Western populations. The magnitude of risks we report here is more akin to other studies conducted in an Asian context. An Indian cohort revealed that 32.5% of women with a history of GDM progressed to T2D when screened at a median of 14 months post-delivery^32^. A Korean study also reported that 17% of women with a history of GDM developed T2D by 4 years post-delivery^17^. Universal GDM screening, an approach increasingly advocated by international authorities^33^ and used in our study population, addresses a common limitation in the literature, as no assumptions were made on the GDM status of those not screened. We and others have shown that selective screening of GDM based on risk factors could result in close to half of the GDM cases being missed^34,35^, and therefore misclassified as non-GDM. Moreover, studies including populations who were only selectively screened during pregnancy, are more likely to be biased towards inclusion of those who already had pre-existing risk factors and thus of a higher baseline metabolic risk; and if they had screened negative and treated as ‘controls’ for assessments of associations between GDM and T2D, the impact of GDM on T2D development could then be underestimated due to dilution of contrast.

Previous studies have primarily investigated the impact of pre-pregnancy weight, GWG and post-delivery weight retention cross-sectionally at specific points in time on the development of T2D^12,16–18^. A strength of our study is that we considered weight status of a woman from pre-pregnancy through postpartum longitudinally to assess their combined influence on the development of post-delivery dysglycaemia, in addition to GDM status.

Our result is in accordance with another Asian study, which showed that post-delivery weight retention or gain during 4 years of follow-up adjusted for pre-pregnancy BMI and last post-delivery follow-up BMI was associated with an increased risk of T2D in women with a history of GDM^17^. However, in our study we also demonstrated that post-delivery weight retention or gain even without a history of GDM was associated with an increased risk of dyslgycaemia 4–6 years post-delivery. In our population, GWG had limited implications for development of post-delivery dysglycaemia. A recent meta-analysis assessing effectiveness of lifestyle interventions for T2D prevention also reported that, among women with GDM, interventions initiated during pregnancy were not effective in reducing the risk of post-delivery T2D; nonetheless, only four studies were included^36^. Our observation that women with inadequate total GWG had a higher risk of T2D may represent reverse causation where some women with metabolic risk factors chose to adopt healthier lifestyle while pregnant, thus gaining less weight.

Lifestyle intervention post-GDM delivery has been shown to be highly effective for the prevention of T2D [pooled RR (95% CI) from ten randomized controlled trials: 0.57 (0.42, 0.78)]^36^. It is also cost-effective, if not cost-saving^37,38^. Using a mathematical model, it was estimated that at least two disability-adjusted life years (DALYs) were averted with proper post-delivery lifestyle management^38^. Among women who were diagnosed with GDM in our study, 43.4% had an abnormal OGTT finding 4–6 years post-delivery and would have benefited from early intervention immediately after delivery. This includes 30.8% who had IFG or IGT, where the progression towards T2D can be prevented or delayed^39,40^.

A common underlying mechanism for GDM development is relative pancreatic insufficiency (β-cell dysfunction)^41^, which is possibly the predominant mechanism in women with normal BMI and among East Asian ethnicities including the Chinese^42^. Increase in insulin resistance is an important normal physiological change with advancing gestation to preserve nutritional supply to the fetus^43^, but the resulting increased pancreatic demands of such maternal adaptation is postulated to accelerate ongoing pancreatic β-cell exhaustion leading to increased T2D risk post-delivery^44^. Alternatively, in OWOB women, excessive adiposity may promote a pro-inflammatory state and insulin resistance, which contribute to both GDM development and later T2D^45^. Both types of mechanisms could thus result in additive effects that may underlie our study observations.

Several limitations to this study need to be acknowledged. The pre-pregnancy weight which was self-reported by the participants at study enrolment may be affected by recall limitation. Nonetheless, the self-reported pre-pregnancy weight and measured booking weight in the GUSTO cohort were highly correlated (ρ = 0·96). BMI is used in this study as a measure of adiposity as commonly used in epidemiological studies. However, we acknowledge that the use of BMI is suboptimal since it does not take differences in body composition into account. The antenatal OGTT at the time of the study visit in 2010 was conducted based on 2 time-points (fasting and 2 h) and GDM diagnosed using the WHO 1999 criteria prior to the release of the IADPSG/WHO 2013 criteria. We had previously reported that if we had adopted the IADPSG/WHO 2013 criteria, without the 1 h glucose measurement, the GDM incidence in GUSTO would have reduced because of the raised threshold for 2 h glucose (and the lack of 1 h glucose), but post-delivery dysglycaemia risk would remain similar^46^. Now, we observed in our sensitivity analysis that had the IADPSG/WHO 2013 criteria been adopted, the trends of a higher risk of developing future dysglycaemia with an increasing number of risk factors (IADPSG-GDM, PDWR, pre-pregnancy OWOB) remained, with some attenuation in effect estimates. This could be due to several reasons that diluted between-group contrasts and BMI effects; the new non-GDM group may have been contaminated by (1) previously diagnosed GDM cases based on WHO 1999 criteria with an intermediate 2 h glucose between 7.8 and 8.4 mmol/L, where healthy lifestyle advice and treatment were given during pregnancy with possible persistent effects post-delivery, and are now reclassified as non-GDM cases, and (2) missed diagnosis of new GDM cases by IADPSG/WHO 2013 criteria (due to lack of data) where there would only have been an isolated abnormal 1 h glucose. Therefore, our results based on the retrospective adoption of the newer criteria should be interpreted with caution. Maternal postnatal OGTT was conducted only at 4–6 years post-delivery and not before, thus the timing of onset of the disease is unknown and a Cox proportional-hazards regression analysis could not be conducted. In addition, of the initial 1165 participants who had pregnancy OGTT conducted, only 59.4% (*n* = 692) went on to have a postnatal OGTT conducted. The modest sample size is a limitation in such modelling work and our findings warrant replication in other cohorts. Furthermore, there could be potential selection bias as the women with both antenatal and postnatal OGTTs were older, tended to have higher educational attainment, and less likely to be nulliparous or had pregnancy-induced hypertension; our observed associations in women who were generally healthier and of higher socio-economic status could be an underestimate for populations with higher underlying risks.

In conclusion, GDM, pre-pregnancy overweight/obesity and post-delivery weight retention independently increase the risk of dysglycaemia at 4–6 years after delivery, although GDM itself poses the highest risk. Overall, the greatest increased risk is observed in women with all three risk factors: a GDM-complicated pregnancy, overweight/obese pre-pregnancy and subsequent substantial post-delivery weight retention. As obesity is a modifiable risk factor, the results of this study support the importance of attaining a healthy weight before pregnancy and avoiding weight retention or gain post-delivery. Unfavourable peri-pregnancy weight status and the high risk of women with a history of GDM progressing to prediabetes and T2D within a relatively short period of time are factors driving further escalation of the epidemic of non-communicable diseases at immense personal, societal, and global health and economic cost. Effective prevention strategies are urgently needed. Pregnancy and post-delivery are times of intensive engagement with healthcare professionals and represent potential opportunities for education and management. However, focusing only on gestational weight gain and interventions during pregnancy alone are not going to have major impact on women’s future health. Instituting preconception care^47^ and effective post-delivery follow-up, especially for those who had GDM, can provide windows of opportunity for promoting long-term health.

## Supplementary Information

Supplementary Information.

## Data Availability

Data are available upon request to the GUSTO team for researchers who meet the criteria for access to confidential data.
