# Supplementary material for: Combined analysis of gestational diabetes and maternal weight status from pre-pregnancy through post-delivery in future development of type 2 diabetes
Source: Sci Rep. 2021 Mar 3;11:5021. doi: 10.1038/s41598-021-82789-x (PMC7930020; doi:10.1038/s41598-021-82789-x)
Supplement: Supplementary file 1 — Supplementary Information. [file 41598_2021_82789_MOESM1_ESM.docx]

**Combined analysis of gestational diabetes and maternal weight status from pre-pregnancy through post-delivery in future development of type 2 diabetes**

**Short running title:** Gestational diabetes, BMI change and post-delivery dysglycaemia

Ling-Wei CHEN, PhD^1*^, Shu E SOH, PhD^1,2*^, Mya-Thway TINT, PhD^1,3^, See Ling LOY, PhD^4,5^, Fabian YAP, FRCPCH ^5,6^, Kok Hian TAN, FRCOG^5,7^, Yung Seng LEE, FRCPCH^1,2,8^, Lynette Pei-Chi SHEK, FAAAAI ^1,2,8^, Keith M. GODFREY, FMedSci^9^, Peter D. GLUCKMAN, FRS^1,10^, Johan G. ERIKSSON, DMSc^1,3,11,12^,Yap-Seng CHONG, MD^1,3^, Shiao-Yng CHAN, PhD^1,3^

^1^Singapore Institute for Clinical Sciences, Agency for Science, Technology and Research, 30 Medical Drive, Singapore 117609

^2^Department of Paediatrics, Yong Loo Lin School of Medicine, National University of Singapore, 1E Kent Ridge Road, Singapore 119228

^3^Department of Obstetrics & Gynaecology, Yong Loo Lin School of Medicine, National University of Singapore, 1E Kent Ridge Road, Singapore 119228

^4^Department of Reproductive Medicine, KK Women’s and Children Hospital, Singapore, Singapore

^5^Duke-National University of Singapore Graduate Medical School, 8 College Road, Singapore 169857

^6^Department of Pediatric Endocrinology, KK Women’s and Children’s Hospital, 100 Bukit Timah Road, Singapore 229899

^7^Department of Maternal Fetal Medicine, KK Women’s and Children’s Hospital, 100 Bukit Timah Road, Singapore 229899

^8^Khoo Teck Puat- National University Children’s Medical Institute, National University Health System, 1E Kent Ridge Road, Singapore 119228

^9^MRC Lifecourse Epidemiology Unit & NIHR Southampton Biomedical Research Centre, University of Southampton & University Hospital Southampton NHS Foundation Trust, Tremona Road, SO16 6YD, Southampton, UK

^10^Liggins Institute, University of Auckland, 85 Park Rd, Grafton, Auckland 1023, New Zealand

^11^Department of General Practice and Primary Health Care, University of Helsinki, Haartmaninkatu 8, 00290 Helsinki, Finland

^12^Folkhälsan Research Center, Topeliusgatan 20, 00250 Helsinki, Finland

*****Ling-Wei Chen and Shu-E Soh are co-first authors

**Corresponding Author**

Shiao Chan, Postal Address: Department of Obstetrics and Gynaecology, Yong Loo Lin School of Medicine, NUHS Tower Block, Level 12, 1E Kent Ridge Road, Singapore 119228; Phone: (65) 6772 2672; Email: [obgchan@nus.edu.sg](mailto:paesse@nus.edu.sg)

**Supplemental Table 1** Criteria for impairment in glucose regulation during pregnancy and post-delivery

|  | **Fasting plasma glucose, mmol/L** |  | **2-hour plasma glucose, mmol/L** | **Diagnostic Criteria** |
| --- | --- | --- | --- | --- |
| **Antenatal OGTT^1^** | | | | |
| **Gestational diabetes mellitus, GDM** | ≥7·0 | **OOR** | ≥7·8 | World Health Organisation (WHO), 1999^15^ |
| **Postpartum OGTT 4-6 years after delivery^2^** | | | | |
| **Type 2 diabetes mellitus, T2D** | ≥7·0 | **OOR** | ≥11·1 | WHO, 2006^16^ |
| **Impaired glucose tolerance, IGT** | <7·0 | **AAND** | 7·8-11·0 | WHO, 2006^16^ |
| **Impaired fasting glucose, IFG** | 6·1-6·9 | **AAND** | <7·8 | WHO, 2006^16^ |

**^1^**During pregnancy, participants diagnosed with GDM were placed on diet-control, with a proportion treated additionally with insulin.

**^2^**After delivery, the women who were diagnosed with diabetes or impaired glucose regulation at the 4-6 years study visit were referred to out-patient clinics for treatment.

OGTT, oral glucose tolerance test

**Supplemental Table 2** Institute of Medicine recommendations for total weight gain and rate of weight gain during pregnancy, by pre-pregnancy body mass index (BMI) categories

|  | **Recommended total weight gain range (kg)^1^** | **Recommended weight gain rate for 2^nd^ and 3^rd^ trimester (kg/week)^1^** |
| --- | --- | --- |
| **Underweight** | 12.7–18.1 | 0.44–0.58 |
| **Normal Weight** | 11.3–15.9 | 0.35–0.50 |
| **Overweight** | 6.8–11.3 | 0.23–0.33 |
| **Obese** | 5.0–9.1 | 0.17–0.27 |

^1^For each pre-pregnancy BMI category, those within the recommended range of total weight gain or weight gain rate were categorized as having adequate gestational weight gain; those with weight gain below the lower boundary of the range were categorized as having inadequate gestational weight gain; those with weight gain above the upper boundary of the range were categorized as having excessive gestational weight gain.

**Supplemental Figure 1** Participants flow chart and data availability

Recruited (*n*= 1450)

Completed antenatal OGTT (*n*= 1165)

Data availability of other relevant information required to determine weight status in those who completed both antenatal and post-delivery OGTT (*n*= 692):

*Pre-pregnancy BMI (n=* 644*)*

*Total pregnancy weight gain (n=* 616*)*

*Postpartum weight retention at month 18 (n=* 509*)*

*Postpartum weight retention at year 4 (n= 574)*

Reached 26-28 weeks’ study visit (*n*= 1239)

Completed post-delivery (Year 4-6) OGTT (*n*= 692)

Multiple pregnancy (*n*= 10)

Lost to follow-up (*n*= 201)

Did not do antenatal OGTT (*n*= 74)

Did not do post-delivery OGTT at year 4-6 (*n*= 473)

OGTT, oral glucose tolerance test

**Supplemental Table 3** Comparison of characteristics for participants with both antenatal and postnatal OGTT with participants with only antenatal OGTT conducted

|  | Only antenatal OGTT | Both antenatal and postnatal OGTT | *P*-value |
| --- | --- | --- | --- |
|  | *n*=473 | *n*=692 |  |
| Ethnicity |  |  | 0.90 |
| Chinese | 268 (56.7%) | 397 (57.4%) |  |
| Malay | 116 (24.5%) | 172 (24.9%) |  |
| Indian | 89 (18.8%) | 123 (17.8%) |  |
| Highest educational attainment |  |  | 0.054 |
| No education/Primary/Secondary | 160 (34.6%) | 194 (28.3%) |  |
| Post-secondary/Pre-university | 158 (34.1%) | 241 (35.1%) |  |
| University | 145 (31.3%) | 251 (36.6%) |  |
| Age at delivery, years | 30.3 (5.1) | 31.8 (5.0) | <0.001 |
| Parity in index pregnancy |  |  | 0.040 |
| Nulliparous | 213 (49.7%) | 300 (43.4%) |  |
| Parous | 216 (50.3%) | 392 (56.6%) |  |
| Family history of diabetes |  |  | 0.80 |
| No | 324 (70.1%) | 472 (69.4%) |  |
| Yes | 138 (29.9%) | 208 (30.6%) |  |
| Insulin treatment for GDM |  |  | 0.96 |
| No | 466 (98.5%) | 682 (98.6%) |  |
| Yes | 7 (1.5%) | 10 (1.4%) |  |
| Hypertension before index pregnancy |  |  | 0.86 |
| No | 467 (98.7%) | 684 (98.8%) |  |
| Yes | 6 (1.3%) | 8 (1.2%) |  |
| Pregnancy-induced hypertension in index pregnancy |  |  | 0.047 |
| No | 402 (92.2%) | 658 (95.1%) |  |
| Yes | 34 (7.8%) | 34 (4.9%) |  |
| Hypertension after index pregnancy |  |  | 0.57 |
| No | 423 (97.0%) | 667 (96.4%) |  |
| Yes | 13 (3.0%) | 25 (3.6%) |  |
| Maternal smoking during pregnancy |  |  | 0.46 |
| No | 445 (95.5%) | 661 (96.4%) |  |
| Yes | 21 (4.5%) | 25 (3.6%) |  |
| Any breastfeeding beyond 6 months |  |  | 0.17 |
| No | 206 (64.0%) | 394 (59.4%) |  |
| Yes | 116 (36.0%) | 269 (40.6%) |  |
| Pre-pregnancy maternal BMI | 22.6 (4.5) | 22.8 (4.4) | 0.44 |
| Pregnancy maternal BMI (booking) | 23.6 (4.8) | 23.7 (4.7) | 0.66 |
| Pregnancy maternal BMI (26-28 wks) | 26.0 (4.5) | 26.2 (4.4) | 0.38 |
| Pregnancy maternal BMI (last antenatal visit) | 28.2 (4.6) | 28.2 (4.4) | 0.95 |
| Postpartum maternal BMI (18 months post-delivery) | 23.9 (4.8) | 24.1 (4.7) | 0.58 |
| Postpartum maternal BMI (4 years post-delivery) | 24.1 (4.6) | 24.7 (5.2) | 0.20 |

OGTT, oral glucose tolerance test; wks, weeks’ gestation

**Supplemental Figure 2** Relative risk of dysglycaemia at 4-6 years post-delivery according to combinations of peri-pregnancy risk factors (with GDM diagnosed based on partial IADPSG criteria)


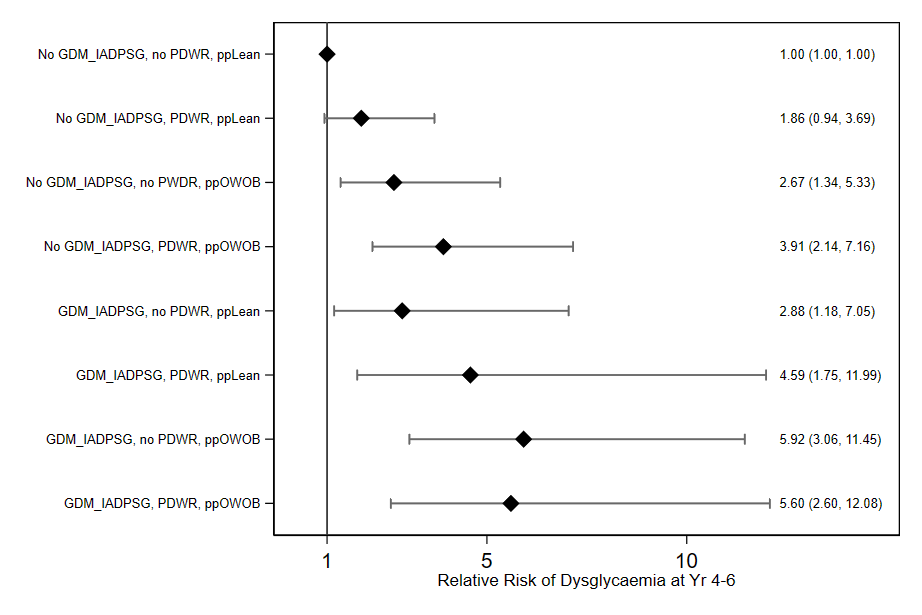


The diamonds and capped lines represent point estimates and 95% confidence intervals, respectively, of the relative risk of having dysglycaemia at 4-6 years post-delivery according to combinations of peri-pregnancy risk factors. Reference group comprised participants without any of the risk factors.

GDM, gestational diabetes mellitus; IADPSG, International Association of Diabetes and Pregnancy Study Groups; PDWR, post-delivery weight retention (≥5 kg) at year 4; ppLean, pre-pregnancy lean (BMI <23 kg/m^2^); ppOWOB, pre-pregnancy overweight or obese (BMI ≥23 kg/m^2^)
